# The fecal bacterial microbiome of the Kuhl’s pipistrelle bat (*Pipistrellus kuhlii)* reflects landscape anthropogenic pressure

**DOI:** 10.1186/s42523-023-00229-9

**Published:** 2023-02-04

**Authors:** Lourdes Lobato-Bailón, Manuel García-Ulloa, Andrés Santos, David Guixé, Jordi Camprodon, Xavier Florensa-Rius, Raúl Molleda, Robert Manzano, Maria P. Ribas, Johan Espunyes, Andrea Dias-Alves, Ignasi Marco, Lourdes Migura-Garcia, Jaime Martínez-Urtaza, Oscar Cabezón

**Affiliations:** 1 Wildlife Conservation Medicine Research Group (WildCoM), Departament de Medicina i Cirurgia Animals, Universitat Autònoma de Barcelona, 08193 Bellaterra, Catalonia Spain; 2 Departament de Genètica i Microbiologia, Universitat Autònoma de Barcelona, 08193 Bellaterra, Catalonia Spain; 3 Centre de Ciència i Tecnologia Forestal de Catalunya, 25280 Solsona, Catalonia Spain; 4 BETA, Universitat de Vic-Universitat Central de Catalunya, 08500 Vic, Catalonia Spain; 5 Asociación Naturalista MUR, 39300 Torrelavega, Cantabria Spain; 6 Unitat Mixta d’Investigació IRTA-UAB en Sanitat Animal, Centre de Recerca en Sanitat Animal (CReSA), Campus de la Universitat Autònoma de Barcelona (UAB), 08193 Bellaterra, Catalonia Spain; 7 IRTA, Programa de Sanitat Animal, Centre de Recerca en Sanitat Animal (CReSA), Campus de la Universitat Autònoma de Barcelona (UAB), 08193 Bellaterra, Catalonia Spain

**Keywords:** Anthropogenic disturbance, Chiroptera, Wildlife, Conservation, Health indicator, Nanopore sequencing, MinION, 16S rRNA

## Abstract

**Background:**

Anthropogenic disturbance has the potential to negatively affect wildlife health by altering food availability and diet composition, increasing the exposure to agrochemicals, and intensifying the contact with humans, domestic animals, and their pathogens. However, the impact of these factors on the fecal microbiome composition of wildlife hosts and its link to host health modulation remains barely explored. Here we investigated the composition of the fecal bacterial microbiome of the insectivorous bat Kuhl’s pipistrelle (*Pipistrellus kuhlii*) dwelling in four environmental contexts with different levels of anthropogenic pressure. We analyzed their microbiome composition, structure and diversity through full-length 16S rRNA metabarcoding using the nanopore long-read sequencer MinION™. We hypothesized that the bacterial community structure of fecal samples would vary across the different scenarios, showing a decreased diversity and richness in samples from disturbed ecosystems.

**Results:**

The fecal microbiomes of 31 bats from 4 scenarios were sequenced. A total of 4,829,302 reads were obtained with a taxonomic assignment percentage of 99.9% at genus level. Most abundant genera across all scenarios were *Enterococcus*, *Escherichia/Shigella*, *Bacillus* and *Enterobacter*. Alpha diversity varied significantly between the four scenarios (*p* < 0.05), showing the lowest Shannon index in bats from urban and intensive agriculture landscapes, while the highest alpha diversity value was found in near pristine landscapes. Beta diversity obtained by Bray–Curtis distance showed weak statistical differentiation of bacterial taxonomic profiles among scenarios. Furthermore, core community analysis showed that 1,293 genera were shared among localities. Differential abundance analyses showed that the highest differentially abundant taxa were found in near pristine landscapes, with the exception of the family *Alcaligenaceae*, which was also overrepresented in urban and intensive agriculture landscapes.

**Conclusions:**

This study suggests that near pristine and undisturbed landscapes could promote a more resilient gut microbiome in wild populations of *P. kuhlii*. These results highlight the potential of the fecal microbiome as a non-invasive bioindicator to assess insectivorous bats’ health and as a key element of landscape conservation strategies.

**Supplementary Information:**

The online version contains supplementary material available at 10.1186/s42523-023-00229-9.

## Background

The majority of Earth’s ecosystems are dominated by human activity and suffer significant and continuously growing disturbances. Consequently, we are witnessing a global biodiversity crisis in which current species extinction rates far exceed background estimates [1, 2]. In turn, biodiversity sustains many ecosystem services needed by humans, and its loss is entailing clear ecological, public health and economic costs at a global scale [3].

The Order *Chiroptera* contributes to worldwide biodiversity with more than 1400 different species, making up one-fourth of terrestrial mammals [4]. This diverse group of mammals inhabits all continents except Antarctica and is responsible to promote and support ecosystem health by means of pollination, seed dispersal and insect and vector-borne diseases regulations [5]. Nonetheless, it is among the most endangered group of mammals with more than 200 bat species around the world considered threatened by the International Union for the Conservation of Nature [4].

Most of the current research efforts relevant to bat conservation worldwide are linked to trends in species diversity and abundance, and distribution patterns [6–8]. Furthermore, investigation into the threats affecting bat species has focused mainly on climate change, habitat degradation and other related human disturbances such as over-hunting, pollution, or collisions with wind energy turbines [9]. Except for the white nose syndrome, which has given rise to extensive research, very little investigation has been done on bat health in contrast to other endangered species [4, 6]. In Europe, bat populations have declined considerably over the last decades presumably due to multiple factors of anthropogenic origin (e.g., pesticides use in agriculture, wind turbines or habitat loss and fragmentation) [9]. Understanding the factors that contribute to such declines and the differential responses of bat species to habitat disturbance is critical for worldwide bat conservation.

Health can be broadly defined as a state of physical and psychological well-being and the subsequent ability to adapt and cope with changing environment [10]. Measuring the health of a group of individuals or populations, particularly in free-ranging species, can be challenging. Health indices, such as body condition or hematological values, are quantifiable parameters used to refer to the health state of a group of animals or species [10]. A specific framework to assess this particular issue in populations of insectivorous bats around the globe has not yet been developed. Difficulties inherent to this broad taxonomic group are not only the requirement of species-specific knowledge of ecology, anatomy, disease susceptibility and pathology, but also arduous sampling conditions often entailing inaccessible sites or technical training [9, 11, 12].

The bacterial communities that inhabit the gut of all animal species constitute the intestinal bacterial microbiota. The genetic and structural elements (e.g., lipids, proteins) and metabolites (e.g., signaling molecules, inorganic and organic molecules, toxins) produced by these organisms in a specific environment, and their theater of activity, are referred to as the bacterial microbiome [13]. Most of the knowledge about the normal gastrointestinal microbial community of bats comes from traditional microbiological studies [14, 15] and they are particularly focused on the presence of infectious agents with zoonotic potential [16, 17]. Current studies on the metagenomic profiling for fecal bacterial communities of bat species have contributed to broadening the knowledge of species-specific microbial diversity, zoonotic pathogens [18–25], diet and niche adaptation, and evolution [26–28].

Two decades of microbiome studies in a wide range of species suggest that intestinal microbiota may contribute not only to the gut’s health but to the overall host’s immunity [29, 30]. Among other characteristics, species-rich communities in a given microbial system appear to be more resilient and to prevent establishment of exogenous microbes -including pathogens- than species-poor communities [31]. This bacterial richness further promotes a better functioning of the community by resource specializing and, in turn, using limited resources more efficiently [32]. As it happens in macroecosystems [33], the inherent properties of resource specializing and resilience from bacterial species-rich communities may be reflected in the overall health status of the host. Given that the microbiome is composed of a dynamic community of bacteria, it is constantly susceptible to change due to age, diet, environment, and diseases among other factors [34]. Hence, substantial changes in species proportions or richness within the gastrointestinal microbial community of a host may lead to dysbiosis, which has been associated to digestive, neurologic, metabolic, and respiratory affections in mammals [35].

Land-use changes for agricultural use and urbanization can negatively affect wildlife health, especially by altering food availability and diet composition, increasing the exposure to agrochemicals, and increasing the contact with humans, domestic animals, and their pathogens. These factors may disrupt the normal gut microbiota and, in consequence, increase the incidence of pathogens which may contribute to the emergence of diseases [36, 37]. Particularly, agriculture development has been shown to create selective pressure on invertebrates [38] and soil microbes [39] through intensification practices and pesticide use. Their impact on the nutrient cycling of soil and its microbes further affects the food webs of ecosystems [40, 41], which eventually may alter the intestinal microbiome of animals. However, evidence for an association between habitat degradation and gut microbiota changes in bat species has barely been explored [42, 43]. Furthermore, information on bat microbial communities -especially these detected in feces- and their role in bat’s health remain scarce. Understanding the complex relationship between host habitat and fecal bacterial composition could contribute to developing a new operational framework for the assessment of bats’ health which can be applied to guide future conservation decisions.

Here, we contribute to the information gathered worldwide about the fecal microbiome of insectivorous bats by exploring the hypothesis that land-use changes and anthropogenic disturbances could shape differences in the composition of the fecal bacterial microbiome of *P. kuhlii*. Because bats dwelling in intensive agriculture and urban landscapes may have access to a less diverse diet and be more exposed to pesticides and pollutants, we hypothesized that bats residing in near pristine ecosystems would show an increased alpha diversity of fecal samples in comparison with bats dwelling in human-disturbed ecosystems. We also expected to find significant differences in the structure of the fecal bacterial community between the different scenarios.

## Methods

### Study areas

Our study areas were located in Catalonia, in the northeast of the Iberian Peninsula. We selected four environmental contexts with different levels of anthropogenic environmental degradation and separated by a minimum of 10 km from each other (Additional file 1: Figure S1 and Table S1): mature and old-growth forest (D0), extensive farming and agriculture (D1), immature and secondary forest (D2), and urban and intensive agriculture landscape (D3). Mature forests from this study were composed of large trees with abundant suitable roosts for bats and were assumed to be free of human disturbance. The selected youth forest had been under logging pressure and habitat fragmentation for decades and was represented by a less complex vegetation structure than mature forests and scarce suitable roosts for bats. In scenario D1, the main use of the soil was pastureland for bovine grazing and crop fields with traditional pest management and moderate pesticide use. The urban and intensive agriculture landscape selected (D3) was located in the Segrià County, which comprises one of the largest intensive pig industry and intensive agriculture area of Spain, and Europe [44, 45].

### Animals and samples

We selected the Kuhl’s pipistrelle (*Pipistrellus kuhlii*), a sedentary and synanthropic bat species in NE-Spain, as a study model. This species indistinctively inhabits open forests and anthropogenic landscapes from the Mediterranean basin and extends throughout Europe. From what is known so far, the home range of this species is less than 2 km^2^, and foraging sites can beas far as 4.5 km [46]. Its ability to dwell in pristine and altered ecosystems makes this species an interesting model for assessing the impact of anthropogenic disturbance on fecal bacterial composition.

Thirty-one Kuhl’s pipistrelles were captured across the four studied landscapes from mid-July to early September 2021 (Additional file 1: Table S2). Bats were captured using harp traps and mist nets and placed in individual and clean cloth holding bags until sampling. Fecal samples were collected directly from bats or from cloth holding bags using sterile forceps. Samples were individually placed in sterile Phosphate Buffered Saline (PBS) (Lonza, Basel, Switzerland) and immediately deposited in dry ice. Once in the laboratory facilities, they were stored at -80ºC until DNA extraction.

Bats included in this study were individually marked with a circular wing biopsy (3 mm punch) which was used for other research purposes but also allowed us to avoid resampling. All sampling procedures followed the EUROBATS best practices [47] and at least one wildlife veterinarian was present in all the captures in order to guarantee the welfare of the captured individuals. No bats resulted harmed or died during the performance of the study.

### DNA extraction, generation of 16S rRNA gene amplicon sequences and library preparation for next-generation sequencing

Fecal DNA was extracted using QIAamp PowerFecal Pro DNA Kit (Qiagen, Hilden, Germany) following manufacturer’s instructions. Initial DNA of the samples was quantified by Qubit Fluorometric Quantification High Sensitivity Assay (Invitrogen, California, USA). 16S rRNA was selectively amplified from genomic DNA by the polymerase chain reaction (PCR) according the SQK-RAB201 Nanopore Kit using universal bacterial primers 27F (5′-AGAGTTTGATCCTGGCTCAG-3′) and 1492R (5′-GGTTACCTTGTTACGACTT-3′), enabling the amplification of approximately 1500 bp of the 16S rRNA gene. PCR amplification was performed in 50 µl of PCR mix comprising 25 µl mix reaction buffer 2 × (LongAmp Taq 2X master mix, New England Biolabs); 14 µl of ultra-pure water; 1 µl of each primer 10 µM; and 10 µl of DNA. The temperature and cycling conditions were as follows: first, preheating at 95 °C for 1 min; then 25 cycles at 95 °C for 20 secs; 55 °C for 30 secs; 75 °C for 2 min; and a final incubation at 65 °C for 5 min. Library construction was performed using the Rapid 16S Amplicon Barcoding Kit (SQK-RAB201) from Oxford Nanopore Technologies (ONT, Oxford, United Kingdom). Two sequencing runs of 20 and 11 multiplexed samples were carried out on a MinION sequencer (ONT) using a brand new R9.6 flow cell.

### Data analysis

All 16S rRNA sequences were obtained by the MinKNOW suite [48] and basecalled with Guppy 3.0. (ONT). Reads were filtered by length (> 1500 bp) and quality (> 10) using NanoFilt 1.1.0 [49]; adapters and barcodes were trimmed with qcat-1.1.0 (ONT). Taxonomic assignment at genus level was carried out with Centrifuge 10.3-beta [50], using Silva 132 database [51] based on a 95% of identity threshold. Afterwards, taxa with single read counts were removed. In addition, low count filter was set to a minimum read count of 4 with a 20% prevalence in the samples. Finally, filtered data was normalized using total sum scaling (TSS). Plots and analysis of microbiomes structure and diversity were made with Pavian-0.3 [52] and MicrobiomeAnalyst [53].

### Statistical analysis

Alpha diversity of each sample was estimated by the Shannon index. Differences between alpha diversity indices were assessed using the Kruskal–Wallis test [54] with a significance threshold set at *p* < 0.05. Additionally, beta-diversity was determined using Bray–Curtis distances and localities were compared using the nonparametric analysis of similarities (ANOSIM) test [55]. Regarding the study of differential taxa among scenarios, a Linear Discriminant Analysis Effect Size (LEfSe) [55] algorithm with a LDA effect size threshold of 2 (on a log_10_ scale) was applied at phylum, family, and genus levels. Moreover, core (taxa shared by 100% of samples), accessory (taxa shared by samples from 2 or 3 scenarios) and exclusive microbiomes (taxa found exclusively in one scenario) were identified using the R package vegan [56]; and the Venn diagram was obtained using the VennDiagram package [57]. Abundance threshold for the core microbiome analysis was set to 0.01. Random effects of sex and locality on alpha and beta diversity were tested with Kruskal–Wallis and a Permutational Mantel test (9999 permutations) based on Spearman’s rank correlation rho, respectively. All statistical analyses were performed using R version 4.1.3 [58].

## Results

The entire 16S gene (≈ 1.5 kb) from the fecal microbiomes of 31 bats was sequenced, for which a total of 4,829,302 reads (7.2 Gb) were obtained with an average of 114,988 ± 43,577 reads per sample. The percentage of taxonomic assignment at the genus level was 99.9% of the total sequences obtained and rarefaction curves of richness against sequence sample size reached asymptotic growth (Additional file 1: Figure S2).

### Taxonomic composition

**Fig. 1:**
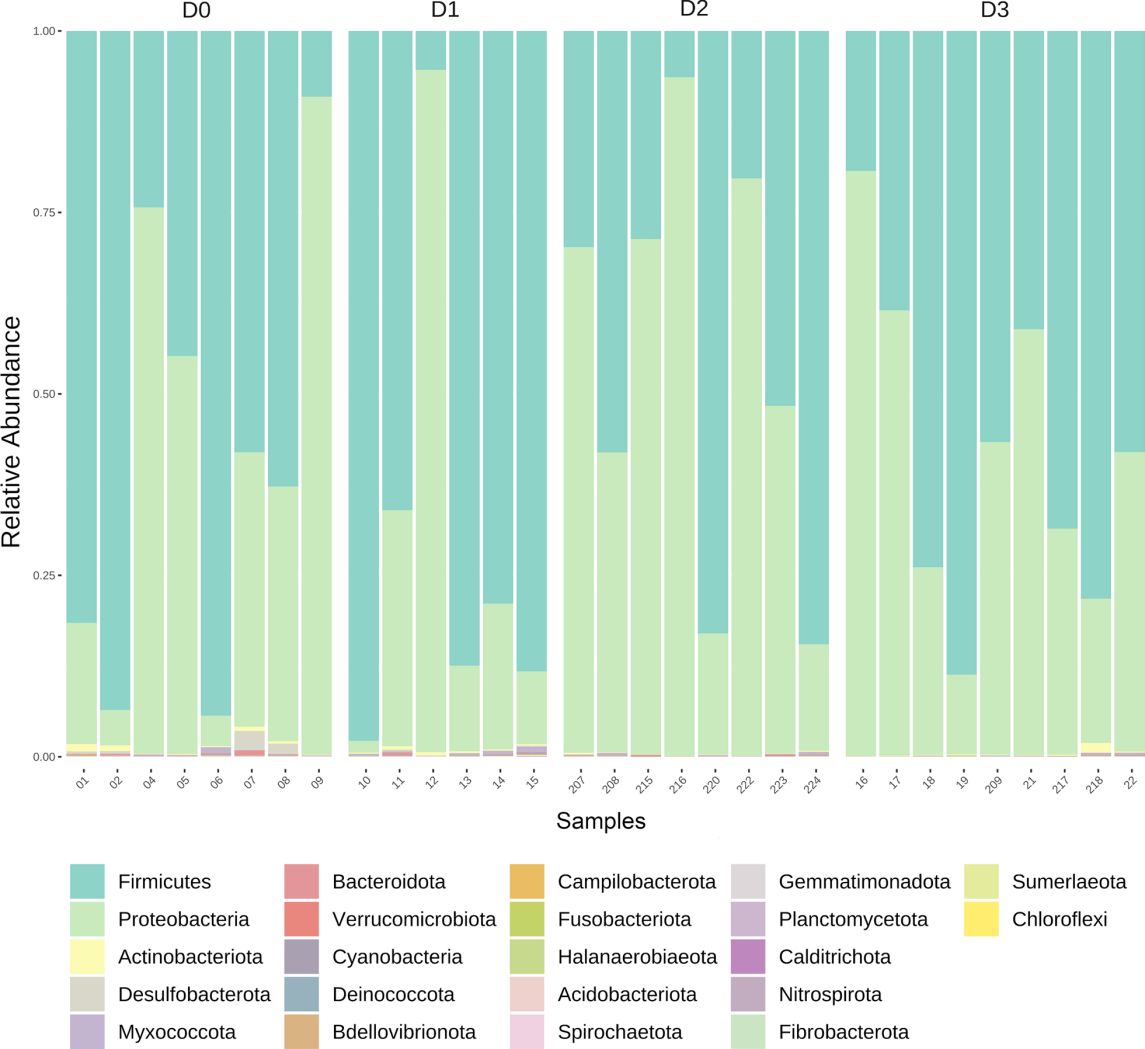
Stacked bar plot of relative abundances of phyla from scenarios D0 (mature and old growth forest), D1 (extensive farming and agriculture), D2 (immature and secondary forest), and D3 (urban and intensive agriculture). Fecal samples are displayed on the bottom and scenarios displayed at the top

*Firmicutes* and *Proteobacteria* were the most abundant phyla in all scenarios (Fig. 1). *Firmicutes* showed a relative abundance of 58.6% ± 31.1 in scenario D0, 70.63% ± 33.7 in scenario D1, 45.3% ± 28.8 in scenario D2 and 58.1% ± 22 in scenario D3. On the other hand, *Proteobacteria* showed an abundance of 39.88% ± 31.9 in scenario D0, 28.32% ± 33.8 in scenario D1, 54.3% ± 29 in scenario D2 and 41.4% ± 22 in scenario D3. *Actinobacteria* was the third most abundant phyla in all the samples. Nevertheless, its relative abundance was < 1% for all scenarios, ranging from 0.09% in scenario D2 to 0.40% in scenario D0.

**Fig. 2:**
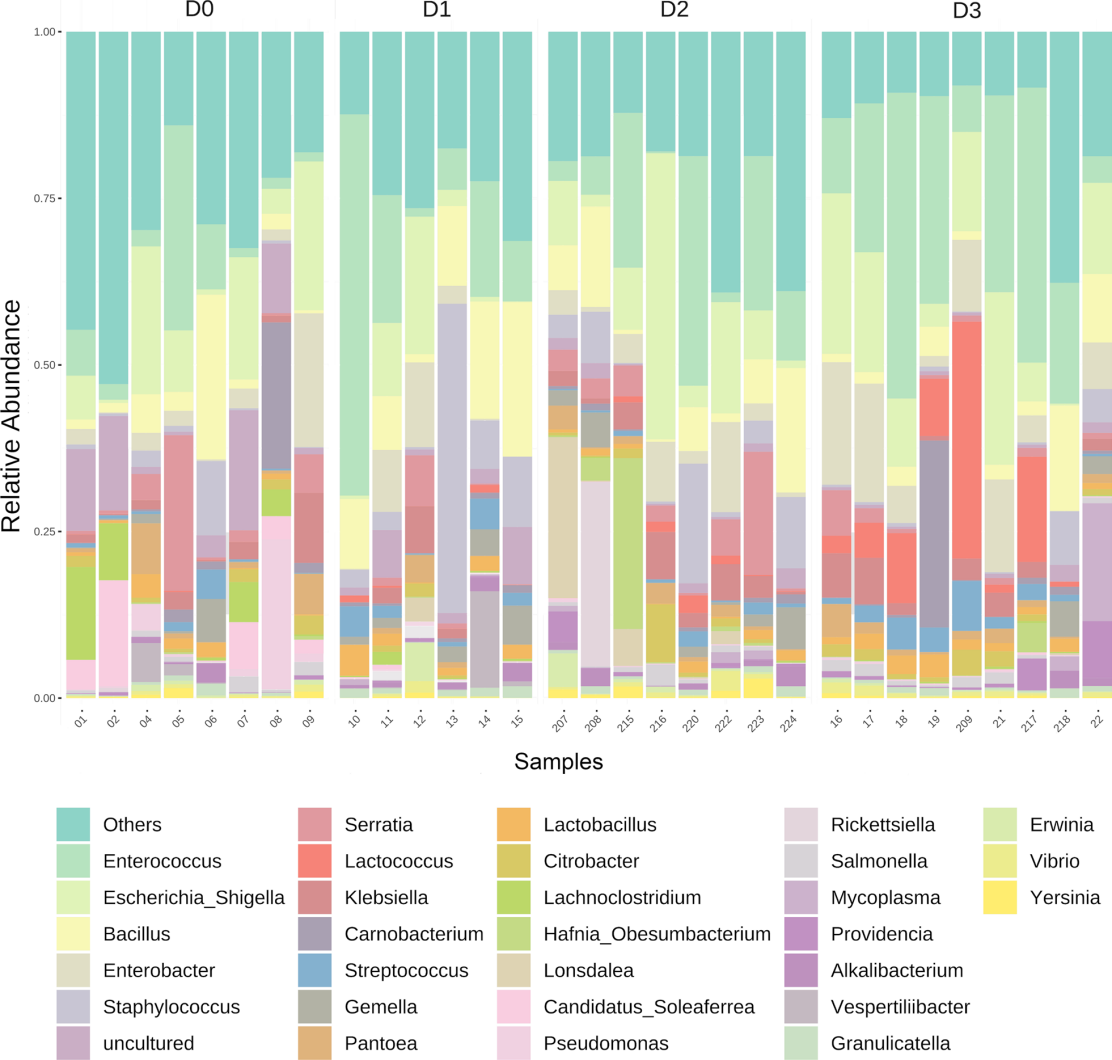
Stacked bar plot of relative abundances of the top 30 most abundant genera from scenarios D0, D1, D2 and D3. Fecal samples are displayed on the bottom and scenarios displayed at the top

Although there was not a strict pattern of relative abundance across all scenarios, *Enterococcus*, *Escherichia/Shigella, Bacillus* and *Enterobacter* were consistently found as the most abundant genera, showing relative abundances (among scenarios) of 15.4% ± 15.1 for *Enterococcus*, 10.5% ± 10 for *Escherichia/Shigella,* 6.7% ± 7 for *Bacillus* and 5.5% ± 6 for *Enterobacter* (Fig. 2). *Enterobacter* displayed both high and low dominance across samples with a range of 0.02% to 20%. The most abundant genera in particular scenarios included *Serratia* (4.56% ± 0.61*)*, *Lachnoclostridium* (4.13% ± 0.25), *Candidatus Soleaferrea* (4.12% ± 0.29), *Pseudomonas (*3.7% ± 0.59)*,* and *Carnobacterium* (3.38% ± 0.56) in D0; *Staphylococcus* (11.7% ± 3), and *Vespertiliibacter* (2.59% ± 3) in D1; *Serratia* (4.23% ± 0.32), *Lonsdalea* (3.58% ± 0.6), *Hafnia* (3.38% ± 0.7), *Ricketsiella* (3.1% ± 0.8) and *Klebsiella* (2.8% ± 0.56) in D2; and *Lactococcus* (8.9% ± 0.12), *Carnobacterium* (3.51% ± 0.85) and *Klebsiella* (3% ± 0.6) in D3.

### Diversity analyses

**Fig. 3:**
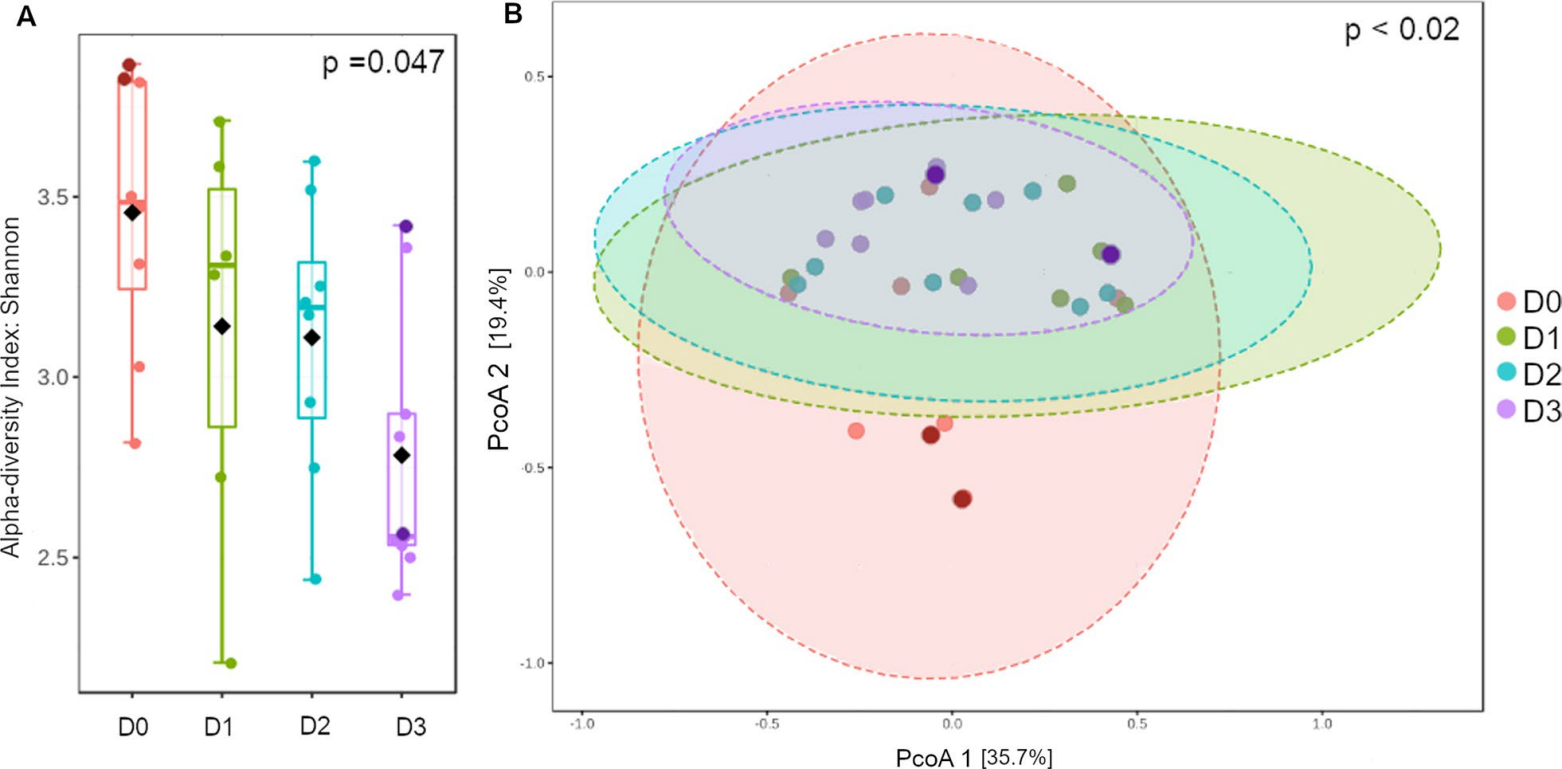
Alpha (**A**) and beta (**B**) diversity measures of the four scenarios: D0, D1, D2 and D3. Boxplot of Shannon’s Diversity Index (Kruskal–Wallis statistic: 7.935; *p* = 0.047) is depicted in panel (**A**). A PcoA of Bray–Curtis distance matrix (ANOSIM’s R: 0.12; *p* < 0.02) is shown in panel (**B**). Different color intensities in dots from scenarios D0 and D3 are used to indicate the different localities sampled within each scenario (Additional file 1: Figure S1 and Table S1 and S2)

Alpha diversity analyses (Fig. 3A) revealed differential diversity patterns for the studied scenarios, showing Shannon index values varying between 2.8 (Scenario D3) and 3.4 (Scenario D0). Scenario D0 showed the highest mean alpha diversity (Shannon’s Index = 3.45 ± 0.38), followed by D1 (3.14 ± 0.57), D2 (3.1 ± 0.38) and D3 (2.7 ± 0.38). Furthermore, Shannon index varied significantly between the four scenarios (H: 7.935; *p* < 0.05), while no significant variance was found between localities (H: 10.45, *p* > 0.05) and the sex of the bats (H: 105, *p* > 0.05). Pair-wise comparisons of Shannon values showed significant differentiation between scenarios D0 and D3 (Additional file 1: Table S3). On the other hand, beta diversity obtained by Bray–Curtis distance showed weak statistical differentiation of community structure across scenarios (Fig. 3B; ANOSIM’s R: 0.12; *p* < 0.02), and pair-wise comparison showed significant differentiation between D0-D3 and D1-D3 (Additional file 1: Table S3). No significant correlation between beta diversity and geographic distance was found (Mantel’s r: 0.013, *p* > 0.05).

**Fig. 4:**
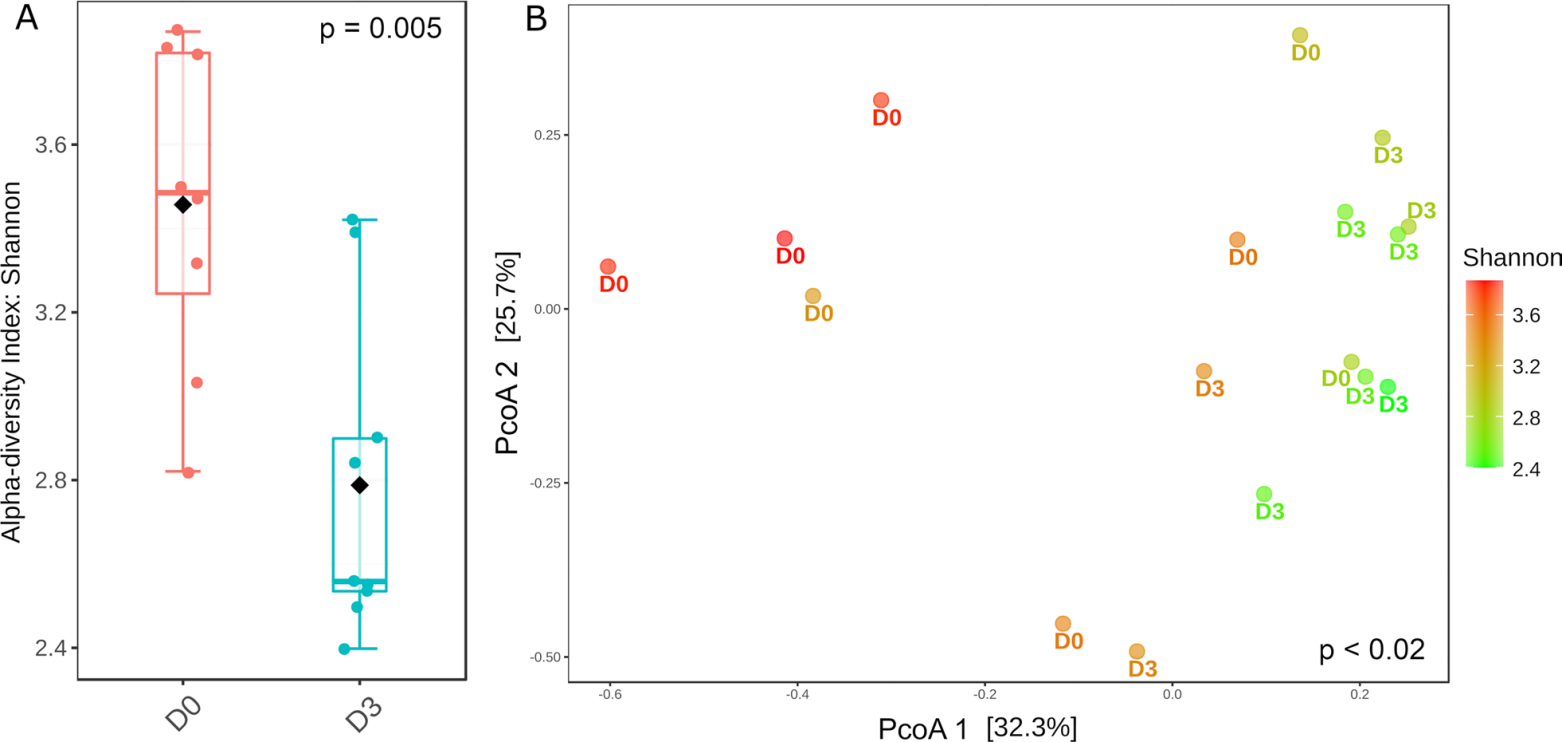
Alpha (**A**) and beta (**B**) diversity measures of scenarios D0 and D3. Shannon’s Diversity Index for D0 and D3 is shown in panel A (Kruskal–Wallis statistic: 64; *p* = 0.005). Bray–Curtis distance matrix (ANOSIM’s R: 0.28; *p* < 0.002) for scenarios D0 and D3 is illustrated in panel (**B**). Alpha diversity is also shown in the ordination plot through a color gradient from green (low value) to red (high value)

Scenarios D0 and D3 displayed the greatest differentiation between each other, both in terms of composition regardless of taxonomic hierarchy (Figs. 1 and 2), and alpha and beta diversities (Fig. 4). Because only scenario D0 included lactating females, we tested whether these samples were influencing the overall results. No significant differentiation in alpha (Kruskal–Wallis statistic: 7; *p* = 1) and beta diversity (ANOSIM’s R: -0.16923; *p* = 0.783) were found between males and females (all lactating) within scenario D0 (Additional file 1: Figure S3).

### Differential abundance analyses

**Fig. 5:**
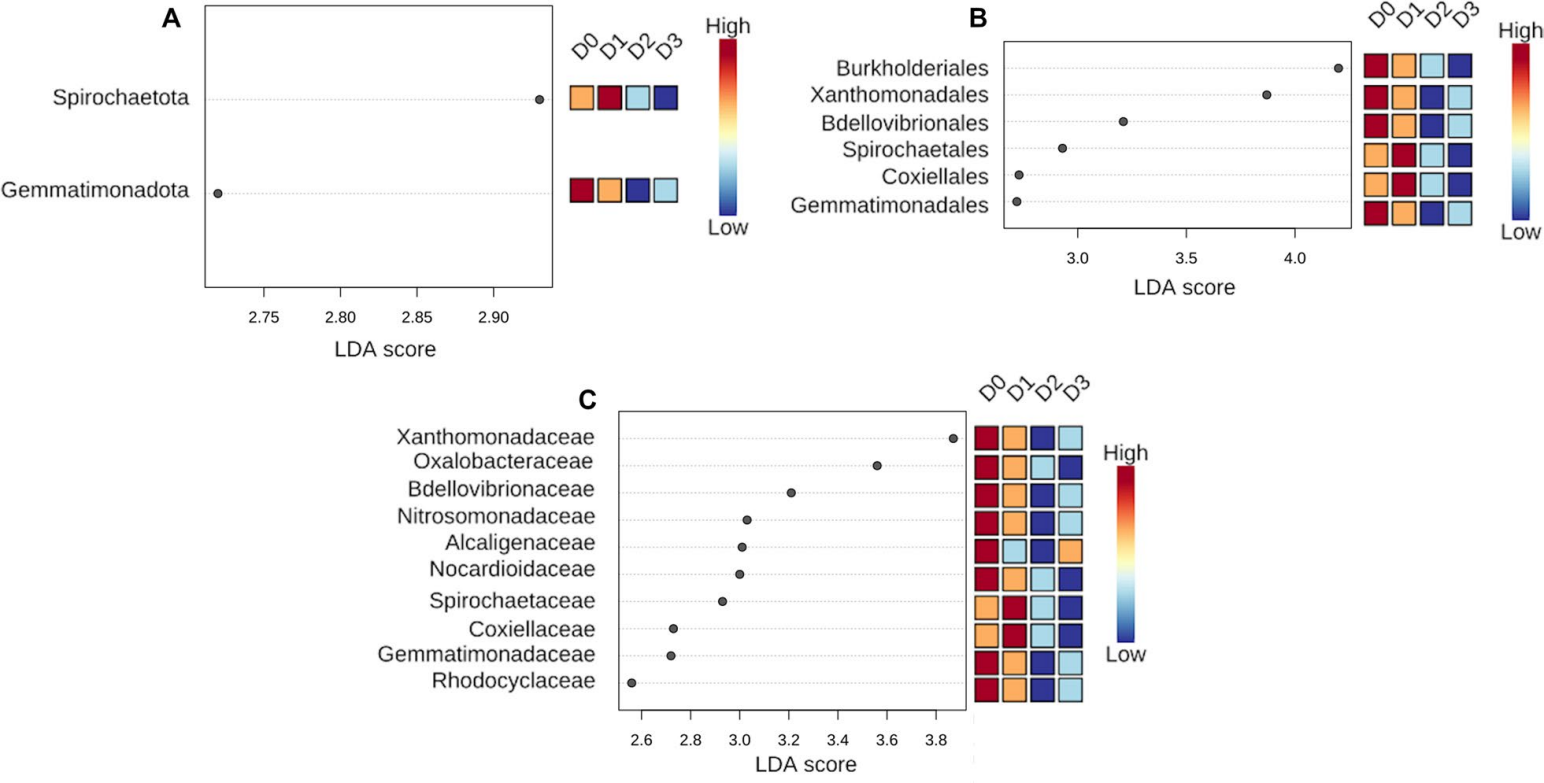
Linear discriminant analysis effect size (LEfSe) of all scenarios. Phylum, order and family taxonomic levels are respectively shown in panels (**A**, **B** and **C**). LEfSe threshold of 2 (on a log_10_ scale) and a significance threshold of 0.05 were set. *p* values were adjusted for false discovery rate (FDR) method

Linear discriminant analyses effect size (LEfSe) of all scenarios showed a distinct pattern of differentially abundant taxa, which were found to be significantly more abundant in scenarios D0 and D1 than D2 and D3 (Fig. 5). Phylum *Gemmatimonadota*; orders *Burkholderiales*, *Xanthomonadales*, *Bdellovibrionales* and *Gemmatimonadales*; and families *Xanthomonadaceae*, *Oxalobacteraceae*, *Bdellovibrionaceae*, *Nitrosomonadaceae*, *Alcaligenaceae*, *Nocardioidaceae*, *Gemmatimonadaceae* and *Rhodocyclaceae* were significantly more abundant in scenario D0. Phylum *Spirochaetota*; orders *Spirochaetales* and *Coxiellales*; and families *Spirochaetaceae* and *Coxiellaceae* were significantly more abundant in scenario D1. With the exception of *Alcaligenaceae*, which appeared in high-to-moderate abundance in scenario D3 and low-to-moderate abundance in D1, all aforementioned taxa were found in low abundance in scenarios D2 and D3. No significant taxa were found at the class and genus levels.

### Core microbiome

**Fig. 6:**
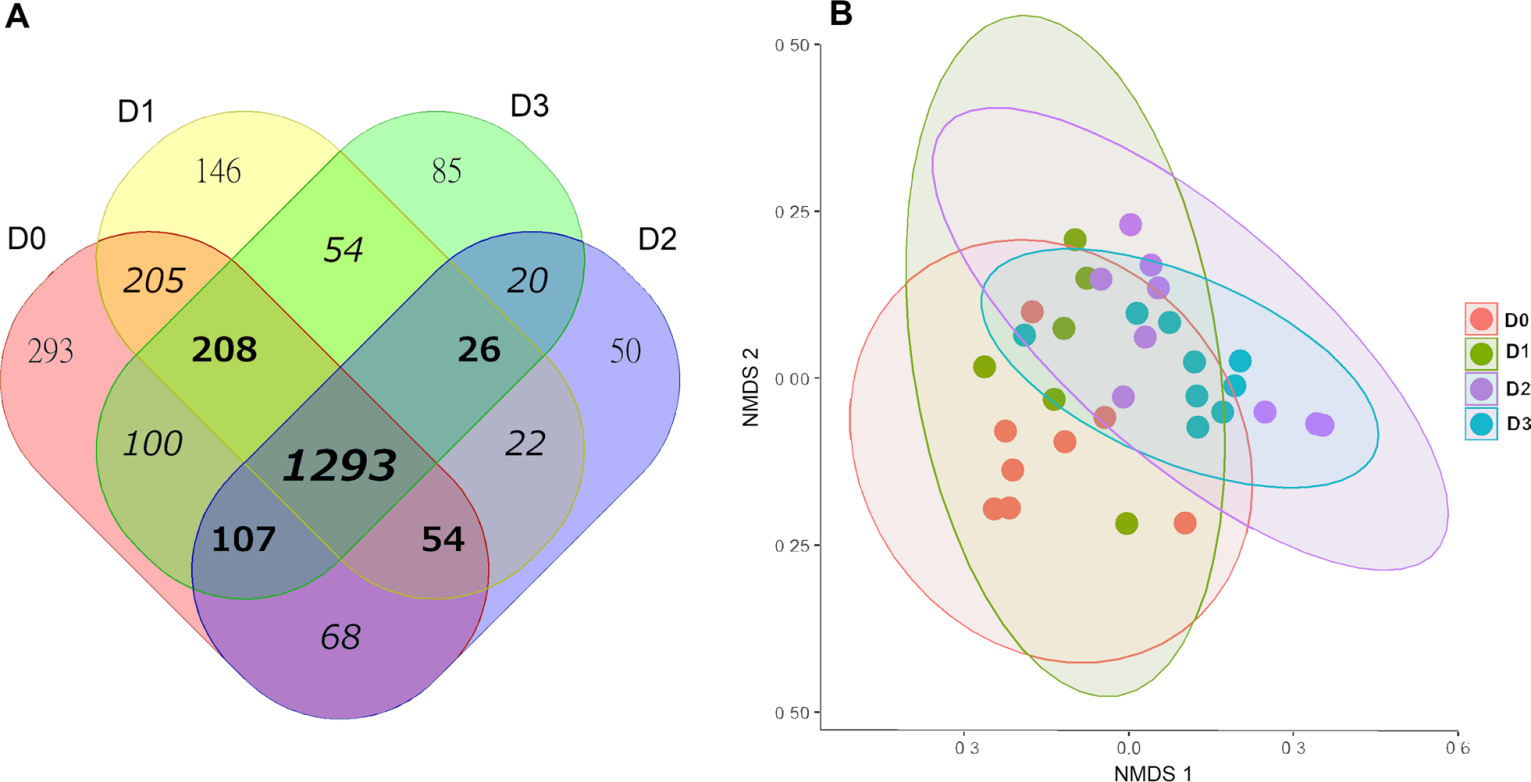
Core community analysis of the four scenarios studied. A Venn Diagram of genera shared across and exclusive to localities is shown in panel (**A**). A Non-metric Multidimensional Scaling plot from a Jaccard distance matrix of presence/absence data is depicted in panel (**B**)

Core community analysis indicated that 1,293 genera were shared by all sites (Fig. 6A). Scenario D0 contained the largest number of exclusive genera (n = 239), while scenario D2 comprised the least (n = 50). A plot of the distance matrix calculated from binary data (presence/absence of genera) roughly depicted two partially overlapping clusters composed of Scenarios D0-D1 and D2-D3 (Fig. 6).

## Discussion

### Similar fecal microbial communities are shared among *P. kuhlii* and other insectivorous bats, regardless of the quality of the environment

According to the literature and supported by our results, chiropteran’s gut microbiota is mainly represented by *Firmicutes* and *Proteobacteria*, while the anaerobic phylum *Bacteroidetes* is underrepresented in bats, but highly dominant in other terrestrial mammals [59]. The fast gastrointestinal transit of bats and their adaptation to flight is believed to be responsible for this *Proteobacteria*-dominated gut microbiota, as also observed in birds [60].

Although the fecal microbiome is strongly influenced by gut microbial communities, guano samples may also be strongly influenced by environmental factors [61]. Nonetheless, the most abundant genera found in fresh feces of *P. kuhlii* from the four scenarios of our study are consistent with the most common taxa identified by NGS in guano samples from different bat species around the globe, particularly *Enterococcus* and *Bacillus* genera [20, 22, 62].

Bacteriological analyses showed the presence of *Escherichia adecarboxylata*, *Citrobacter freundii*, *Klebsiella oxytoca*, *Enterobacter agglomerans*, *E. aerogenes*, *E. gergoviae*, *Proteus vulgaris* and *Streptococcus faecalis* in feces of *P. kuhlii* from Italy [15]. While *Enterobacter*, *Klebsiella*, *Streptococcus*, *Escherichia*, and *Citrobacter* genera were also present in our analyses, different isolation and detection techniques could have contributed to different findings such as the high abundance of *Enterococcus* genera across all our studied scenarios. Since most microbes remain unculturable, using culture-independent analyses in microbiome studies is essential to better understand and characterize bacterial communities and their functions [63]. As demonstrated by Newman et al. in 2018, the observation of a high abundance of the genus *Vespertiliibacter* in scenario D1 from our study would have been neglected by culture-dependent analyses [64]. This *Pasteurellaceae* was isolated for the first time in Germany in the upper respiratory tract of three different bat species of the family *Vespertilionidae*, including one *P. pipistrellus* [65]. More recently, it has also been isolated in guano samples from a maternal colony of *Tadarida brasiliensis* (family *Molossidae*) in New Mexico (US), showing a greater presence on fresh guano samples over surface or subsurface guano samples [63]. However, the role of the genus *Vespertiliibacter* as a commensal or opportunistic pathogen for bats is yet to be elucidated. As a dominant genus in samples from scenario D1, it may be responsible of meaningful functions in the microbiome of these bats.

Despite partial overlapping of fecal microbial communities exists between bats with different trophic strategies [27], diet specialization is generally linked with specific microbiota capable of nutrient assimilation [66]. In this sense, significant differences in microbiome composition between frugivorous, insectivorous and piscivorous bats have been reported by several authors [62, 67–69]. More recently, different bat dietary habits have also been linked to differences in metagenome functions which may be linked to specific metabolic pathways [28]. Several most abundant genera from our study (*Carnobacterium, Serratia, Hafnia, Enterococcus* and *Lonsdalea*) were also found to be significantly abundant in some insectivorous bat species across Europe, China, Israel, Mexico and Costa Rica, compared to piscivorous bat species [68]. Notably, we found that *Serratia*, *Hafnia* and *Lonsdalea* genera were differentially abundant in *P. kuhlii* from scenario D2. Insectivorous bat species from the former study included *Pipistrellus kuhlii*, other vespertilionid and rhinolophid bats, but specific characteristics of the sampling localities were not specified. Moreover, none of the most differentially abundant genera present in piscivorous bats were represented in our study [68], further supporting the current scientific data about the significant impact of trophic guilds on fecal bacterial communities [66].

Additionally, the predominant bacterial genera in fecal samples from scenario D0 were found to particularly overlap with fecal samples of *P. pipistrellus* and other insectivorous bats from the Netherlands, sharing *Carnobacterium*, *Serratia* and *Pseudomonas* as the most abundant genera [17]. Interestingly, we would have anticipated greater similarities between either scenario D1, D2 or D3 since the bats from the latter study were captured in lime-stone mines near villages rather than in near pristine habitats. These counterintuitive results may be explained by the fact that mines are usually located in rural areas and, once mine activities cease, sites tend to be partially restored to pre-mining conditions, with minimal human presence [70]. However, persistent environmental pollutants of different mining activities can also alter the fecal microbiome of bats and their impacts still need to be clarified.

### Intensive agriculture and urban landscapes may be enhancing intestinal bacterial lineages with pollutant-degrading properties

By linear discriminant analyses effect size (LEfSe), differential abundance taxa, including the order *Burkholderiales*, appeared in high abundance in scenario D0. The order *Burkholderiales* constitutes a metabolic and ecologically diverse bacterial linage, which includes human opportunistic bacteria (mostly nosocomial), animal and plant pathogens, bacteria present in wastewater and sludge, and bacteria naturally present in soil, freshwater and sediment [71, 72]. This order comprises the families *Alcaligenaceae*, *Burkholderiaceae*, *Comamonadaceae*, *Oxalobacteraceae*, *Sutterellaceae*, *Sphaerotilaceae* and *Burkholderiales* genera *incertae sedis* [73]. Our results showed that, while *Oxalobacteraceae* and *Alcaligenaceae* were highly abundant in scenario D0, *Alcaligenaceae* was also the only family overrepresented in scenario D3.

Species belonging to the family *Oxalobacteraceae* have shown the ability to invade and persist in different niches, such as plant tissues, Antarctic soil, rivers and lakes, groundwater and contaminated soils among others [72]. Moreover, species belonging to this family have been recovered from patients with clinical disease and are considered mild and opportunistic human pathogens [72]. On the other hand, the family *Alcaligenaceae* includes some well-recognized primary animal and human pathogens such as the genus *Bordetella* [72] and *Taylorella* [74]. Bacteria from the family *Alcaligenaceae* have also been isolated from biogas slurry (genus *Advenella*) [75], activated sludge (genera *Caenimicrobium* and *Pusillimonas*) [76, 77] and other samples from wastewater treatment plants (genera *Parapusullimonas* and *Pigmentiphaga*) [78, 79]. Other bacteria from this family have been isolated from freshwater sources (genus *Parvibium*) [80] or have shown the ability to thrive in different environments such as the genus *Pusillimonas*, isolated from wastewater treatment plants, farm soil and poultry manure among others [77, 81, 82]. Although the ecological function of bacteria shouldn’t be defined solely by their source of isolation, many genera from the family *Alcaligenaceae* have shown the ability to degrade Polychlorobiphenyl (PCB) [83], neonicotinoids [84] and aromatic hydrocarbons [71, 85], pointing out their potential in removing environmental pollutants. The high abundance of *Alcaligenaceae* in bats from scenario D3 may be explained by the gut bacterial community’s response to pesticides and other pollutants exposure, promoting the growth of detoxifying bacteria. The differentially high abundance of this family in scenario D0 in contrast to D1 and D2 may not be so straightforward. Evidence of atmospheric transport and deposition of pollutants into mountain ecosystems (cold-trapping effect) have been reported for several pollutants, including microplastics [86] and semi-volatile organic chemical pollutants [87]. Lles de Cerdanya, one of the sampled localities in scenario D0, is located in a montane area of the southeastern Pyrenees. Therefore, despite being far from major populations or industrial centers, the high elevation of this village may aid the deposition of air chemical pollutants through rain, snow, fog or wind.

The family *Alcaligenaceae* has been described in the fecal microbiome of different bat species [24, 25, 88], regardless of their dietary habits or geographic distribution. Furthermore, this family of proteobacteria was found to be characteristic of Palla’s Long-tongued bats (*Glossophaga soricine*) foraging in natural forests in comparison to those foraging in organic or conventional banana monocultures where pesticides were used [43]. As a highly diverse bacterial linage, further research is needed to understand *Alcaligenaceae*’s functional significance in the gut microbiome of bats.

### Multiple variables may explain why healthier ecosystems support more resilient fecal bacterial microbiomes

We demonstrated that the richness and diversity of the fecal bacterial microbiome of *P. kuhlii* bats were affected by the level of anthropogenic disturbance (Shanon Index; *p* < 0.05), while gender and geographical distance between sampling sites had no significant impacts on the microbiome composition (Mantel test; *p* > 0.05). Variation in gut microbial diversity of bats has been linked to shifts in season (mainly pre- and post-hibernation) and specific reproductive states such as lactation or pregnancy [89, 90]. Three females from scenario D0 were captured during the lactation period, when microbial diversity often increases [88]. However, no significant differences in alpha and beta diversity were found between these lactating females and the rest of the bats from the same scenario, thereby ruling out any potential effect of lactation on the overall microbial diversity.

In our study, *P. kuhlii* inhabiting pristine forests displayed richer fecal bacterial microbiomes than those dwelling in degraded environments such as farmlands and urban landscapes. Defining what represents a healthy gut microbiome has proven difficult, particularly due to the high interindividual variability [91]. However, resistance and resilience to external perturbations are among the crucial characteristics of a healthy microbiome [92, 93] and both are positively affected by high microbial diversity [32]. Accordingly, the higher alpha diversity displayed by feces of Kuhl’s pipistrelles from undisturbed forests may be indicative of a more resilient gut microbiome compared to that of bats dwelling in disturbed environments.

The gut microbiota at the population level is supposed to be evolutionarily selected and to remain relatively stable across individuals of the same species [94]. Nevertheless, environmental factors can mediate bacterial community structure as demonstrated in human and other animal studies. Such is the case of industrialization, which has been related to shifts in the relative abundance of bacterial lineages and reduced microbiota diversity in human populations [95, 96]. Human activities have also modified how wild animals move through the landscape and interact with its biological components [97]. In this sense, shifts in intestinal microbiota composition and diversity from wild animals have been reported as consequences of habitat fragmentation and encroachment [98, 99]. Human-disturbed and fragmented landscapes were also associated with significantly lower alpha diversity in the gut microbiome of Tome’s spiny rats (*Proechimys semispinosus*) [100]. However, when considering habitat fragmentation without additional anthropogenic disturbances such as contact with humans and domesticated animals, no impacts were detected [100]. Similar to our study, Barelli et al*.* [101] showed that colobus monkeys (*Procolobus gordonorum*) inhabiting undisturbed forests from Tanzania had significantly higher alpha diversity than those from disturbed forests. Through functional analyses they concluded that this variation was associated with dietary changes and diversity derived from anthropogenic habitat degradation [101].

The Kuhl’s pipistrelle is considered a selective-opportunist feeder, targeting larger preys over the smaller ones. The most important orders of insects in the diet of *P. kuhlii* are *Coleoptera*, *Diptera*, *Hemiptera*, *Heteroptera*, *Hymenoptera* and *Lepidoptera*. In the Iberian Peninsula, *Diptera* and *Lepidoptera* account for the main representative orders of their diet, and these include several agricultural pests such as *Pectinophora gossypiella* (i.e. cotton pest) [46]. However, as an opportunistic feeder, variation in insect diversity and volumetric representation may be encountered in the diet of Kuhl’s pipistrelles across different types of landscape. Further explorations of *P. kuhlii’s* diet across the different scenarios from this study could provide a better overview of how insects respond to these specific anthropogenic changes and their potential effect on the fecal microbiome of this bat species. Land-use changes, particularly urbanization and agriculture, are probably responsible for the recent decline in insect abundance and biotic homogenization [102–104]. Future research assessing the potential effects of diet homogenization on the health of insectivorous bats may highlight the relevance of specific habitat conservation strategies.

Another consequence of human activities is the release of toxic substances into air, water and land at harmful rates for living beings [105]. Therefore, wildlife that manages to thrive and adapt to urban and agricultural landscapes faces constant or temporary exposure to pollutants and pesticides. A growing body of evidence indicates that pesticides may induce changes in the gut’s microbiota composition and lead to dysbiosis or alteration of the homeostasis in several host species [106]. Insectivorous and frugivorous bats forage equally in plantations -feeding on pests or crops respectively- as well as in forests or natural areas. Alpízar et al. [43] revealed that nectar-feeding bats *Glossophaga soricine* foraging intensively managed banana plantations had a significantly lower alpha diversity than those foraging organic banana plantations, and far lower than bats foraging natural forests. Although a less diverse diet may explain this association, no differences in diet were seen among bats foraging intensively managed plantations and those foraging organic plantations, suggesting that reduction in gut microbiota diversity may be caused by other factors such as the use of pesticides [43]. The use of pesticides in the studied agricultural, logged, and urban landscapes from Catalonia is a common practice (personal observation). Therefore, the lower alpha diversity of Kuhl’s pipistrelles from these scenarios may be further explained by similar factors to those suggested in *G. soricine* [43].

### Fecal bacterial microbiome analysis as a potential health indicator for free-ranging insectivorous bat populations

Significant differences in community structure found between scenarios D0 and D3, which were not explained by geographic distance or different reproductive status, highlight the potential impact of human activities on the fecal microbiome composition of insectivorous bats. Furthermore, we showed that the fecal bacterial richness of wild populations of Kuhl’s pipistrelles decreased along a gradient of increasing urbanization and other anthropogenic disturbances such as the presence of domesticated animals. Due to the fact that loss of microbiota diversity opens up niches for opportunistic pathogens [35], our results suggest that anthropogenic disturbance may modify the gut composition and functionality, and pose a risk to the health of insectivorous bats.

Owing to its non-invasive nature and simplicity of sampling, we propose the fecal bacterial microbiome as a health indicator for free-ranging insectivorous bats. Nevertheless, larger sample sizes, and experimental and longitudinal studies are needed to explore the dynamics of bat’s gastrointestinal microbiome through life and its adaptation processes to environmental disturbances. Future studies should also explore other factors that may modulate differences in microbiome composition such as bat species, seasonality, diet, and reproductive and physiologic status.

## Conclusion

Our study demonstrates a correlation, rather than causal relationship, between anthropogenic pressure and the fecal bacterial microbiome of Kuhl’s pipistrelles. Nonetheless, the inverse correlation found between the level of anthropogenic disturbance and the fecal bacterial microbiome richness and diversity of *P. kuhlii* indicates that near pristine and undisturbed landscapes could promote more resilient gut microbiomes. Studies on physiological parameters for different bat species during their variable and complex life cycle are needed to establish reference values and, in turn, to be able to link microbiome changes to health and disease status.

The results of this study generate new questions about the impact of degraded habitats on the fitness of bats via the microbiome and highlight its potential as a non-invasive bioindicator to assess insectivorous bats’ health. We believe that such studies will, additionally, serve as a key element of landscape conservation strategies.

## Supplementary Information

**Additional file 1. Figure S1.** Map of sampling locations. Localities and their associated scenarios are depicted in the map with colored dots covering the foraging range of *Pipistrellus kuhlii* (4.5km): D0 (mature and old-growth forest), D1 (extensive farming and agriculture), D2 (immature and secondary forest), and D3 (urban and intensive agriculture landscape). **Figure S2.** Rarefaction curves of each sample, separated by scenario. Sequence sample size (number of reads generated) and genus richness are depicted on the X and Y-axis, respectively. **Figure S3.** Comparison of diversity between males and lactating females from scenario D0. No significant differentiation in alpha diversity given by Shannon’s Index (Panel A; Kruskal-Wallis statistic: 7; p = 1) and beta diversity given by Bray-Curtis distance (Panel B; ANOSIM’s R: -0.16923; p = 0.783) were found. **Table S1.** Environmental information of the scenarios selected. **Table S2.** Biological data of sampled bats, time and location of sampling. **Table S3. **Pair-wise comparison of alpha and beta diversities between scenarios. Significant results are signaled with *. **Table S4.** Biosample accession numbers for datasets obtained in this study.

## Data Availability

The dataset supporting the conclusions of this article is available in the GenBank repository, (https://www.ncbi.nlm.nih.gov/biosample/). Biosample numbers are available in Additional file 1: Table S4.
