# Supplementary material for: The fecal bacterial microbiome of the Kuhl’s pipistrelle bat (Pipistrellus kuhlii) reflects landscape anthropogenic pressure
Source: Anim Microbiome. 2023 Feb 4;5:7. doi: 10.1186/s42523-023-00229-9 (PMC9898988; doi:10.1186/s42523-023-00229-9)
Supplement: Supplementary file 1 — Additional file 1. Figure S1. Map of sampling locations. Localities and their associated scenarios are depicted in the map with colored dots covering the foraging range of Pipistrellus kuhlii (4.5km): D0 (mature and old-growth forest), D1 (extensive farming and agriculture), D2 (immature and secondary forest), and D3 (urban and intensive agriculture landscape). Figure S2. Rarefaction curves of each sample, separated by scenario. Sequence sample size (number of reads generated) and genus richness are depicted on the X and Y-axis, respectively. Figure S3. Comparison of diversity between males and lactating females from scenario D0. No significant differentiation in alpha diversity given by Shannon’s Index (Panel A; Kruskal-Wallis statistic: 7; p = 1) and beta diversity given by Bray-Curtis distance (Panel B; ANOSIM’s R: -0.16923; p = 0.783) were found. Table S1. Environmental information of the scenarios selected. Table S2. Biological data of sampled bats, time and location of sampling. Table S3. Pair-wise comparison of alpha and beta diversities between scenarios. Significant results are signaled with *. Table S4. Biosample accession numbers for datasets obtained in this study. [file 42523_2023_229_MOESM1_ESM.docx]

**Additional file 1**

**Supplementary figures**

**
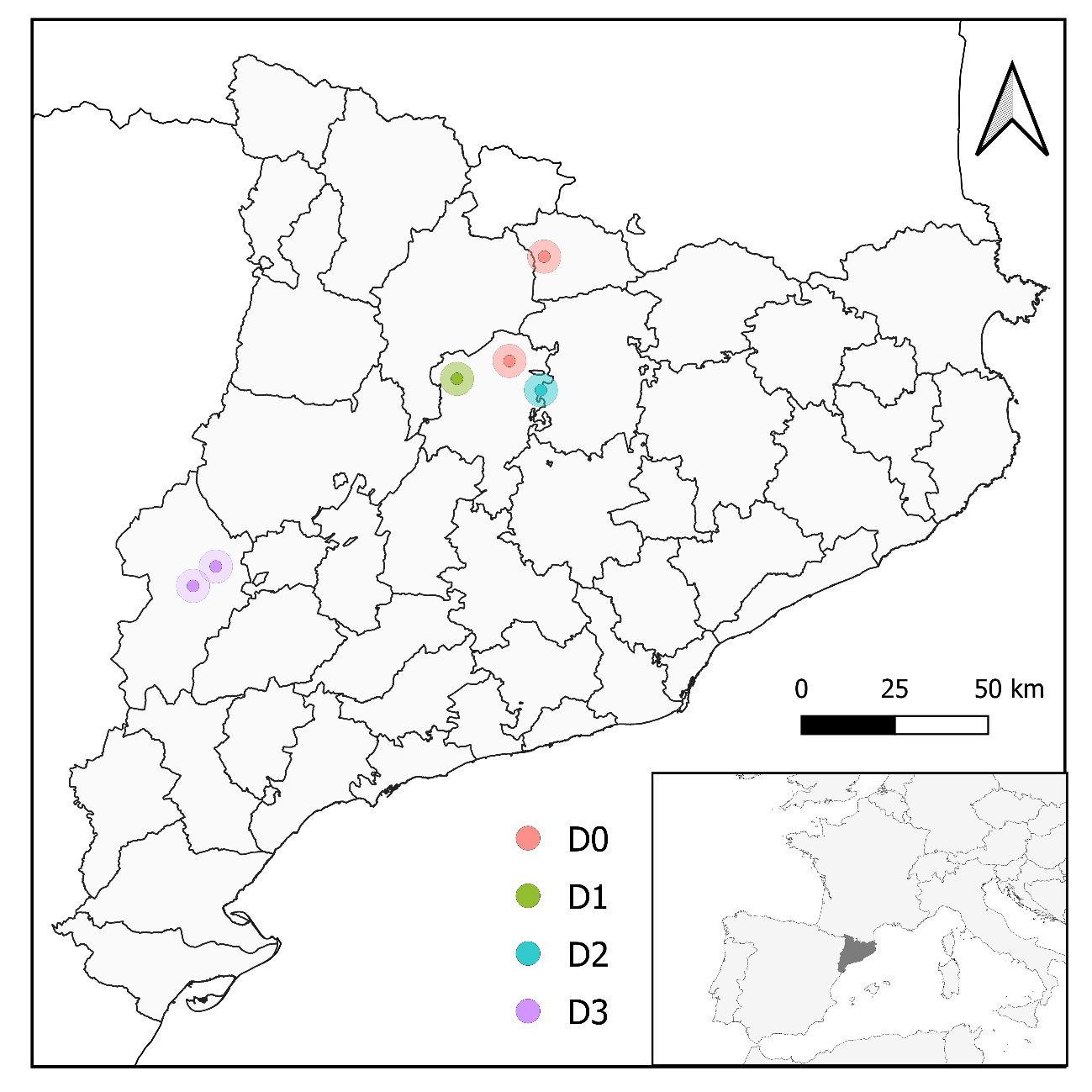
**

**Figure S1. Map of sampling locations. Localities and their associated scenarios are depicted in the map with colored dots covering the foraging range of *Pipistrellus kuhlii* (4.5km): D0 (mature and old-growth forest), D1 (extensive farming and agriculture), D2 (immature and secondary forest), and D3 (urban and intensive agriculture landscape).**

**
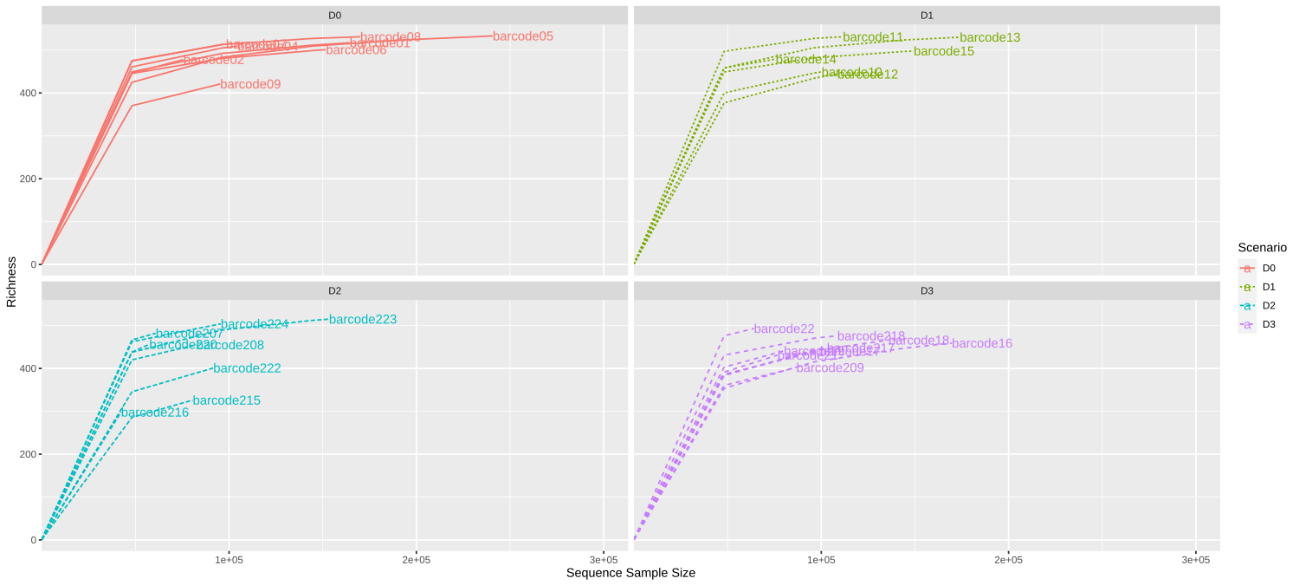
**

**Figure S2. Rarefaction curves of each sample, separated by scenario. Sequence sample size (number of reads generated) and genus richness are depicted on the X and Y-axis, respectively.**

**
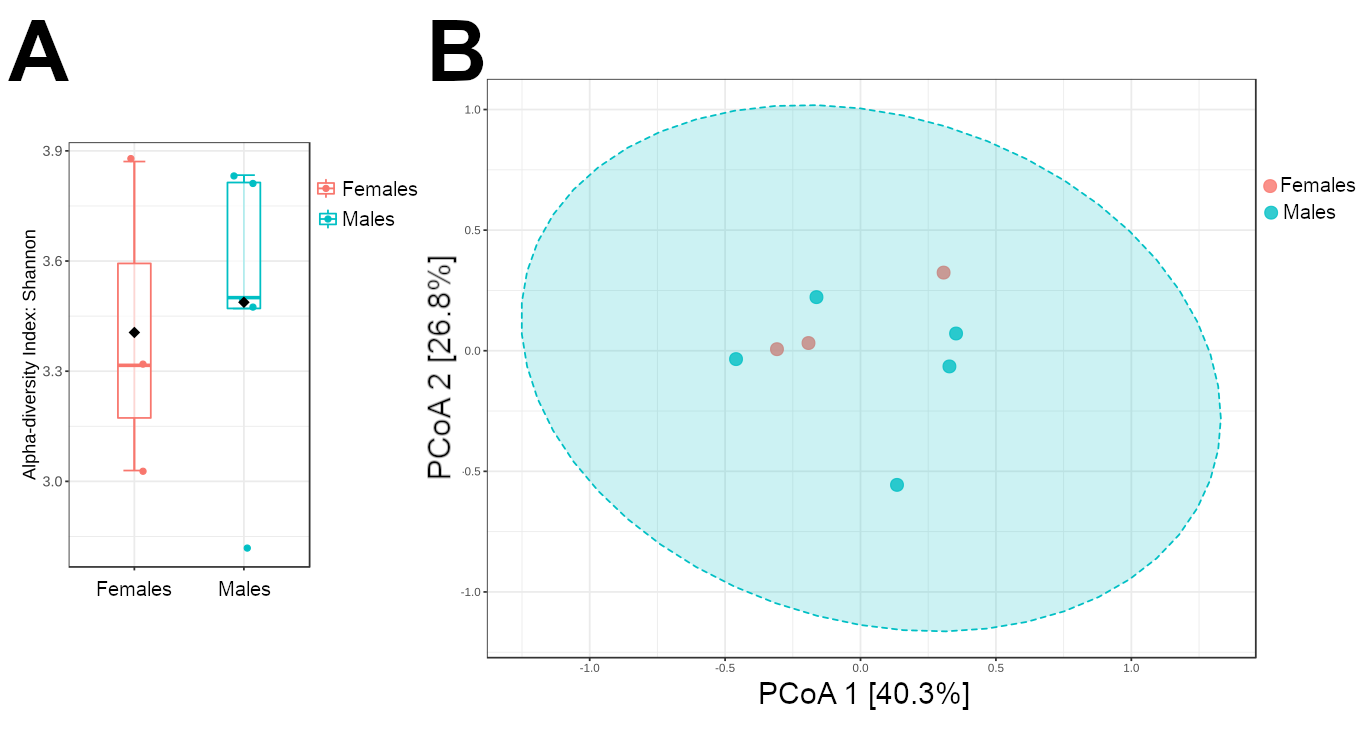
**

**Figure S3. Comparison of diversity between males and lactating females from scenario D0. No significant differentiation in alpha diversity given by Shannon’s Index (Panel A; Kruskal-Wallis statistic: 7; p = 1) and beta diversity given by Bray-Curtis distance (Panel B; ANOSIM’s R: -0.16923; p = 0.783) were found.**

**Supplementary tables**

**Table S1. Environmental information of the scenarios selected.**

| **Scenario** | **D0** | | **D1** | **D2** | **D3** | |
| --- | --- | --- | --- | --- | --- | --- |
| **Locality** | Lles de  Cerdanya | Sant Llorenç de Morunys | Lladurs | L'Espunyola | Lleida city | Lleida outskirts |
| District | Baixa Cerdanya | Solsonès | Solsonès | Berguedà | Segrià | Segrià |
| X,Y coordinates | 42.388056,1.687222 | 42.136111,1.580833 | 42.09256,1.41260 | 42.06748,1.41260 | 41.63102,0.64231 | 41.57864,0.64231 |
| ^1^Elevation (m) | 1471 | 925 | 834 | 803 | 155 | 130 |
| ^1^Surface area (km²) | 102.79 | 4.34 | 128.03 | 35.46 | 212.30 | 10.46* |
| ^1^Population in year 2021  (total hab.) | 288 | 985 | 187 | 258 | 140,080 | 2,224* |
| ^1^Declared industrial waste  in tonnes (last year of declaration) | 0.01 (2019) | 3 (2020) | 1 (2014) | 10 (2015) | 24,607.22 (2020) | 1 (2020)* |
| ^2^Main land use/cover | Forest and high mountain meadows | Mature forest (±meadows) | Extensive farming and immature forest | Forest for logging and extensive farming | Intensive agriculture and urban land | Intensive agriculture and urban land |
| ^2^Main tree cover | Red pine and oak | Red pine and oak and Holm oak | Pine and Holm oak | Pine (±Holm oak) | NA | NA |
| ^1^Main livestock | NA | NA | Cattle, pig and poultry | Pig and poultry | Pig, poultry, cattle and sheep | Pig and poultry* |
| ^3^Average 2021 temperature (0ºC) | 8 | 10 | 12 | 12 | 15 | 15 |
| ^3^Average 2021 minimum temperature (0ºC) | 2.5 | 5 | 5 | 7.5 | 7.5 | 7.5 |
| ^3^Average 2021 maximum temperature (0ºC) | 12.5 | 12.5 | 17.5 | 15 | 22.5 | 22.5 |
| ^3^Cumulative rainfall in 2020 (mm) | 700 | 550 | 400 | 550 | 250 | 250 |
| ^3^Average frost days in 2020 | 100 | 100 | 50 | 50 | 25 | 25 |
| ^3^Average snow days in 2021 | 20 | 10 | 3 | 5 | 1 | 1 |
| ^4^Closest Air Quality station (district) | Bellver de Cerdanya | Berga | Berga | Berga | Lleida city | Lleida city |
| ^4^Average PM2,5 detection  in 2021 (ug/m3) | 17.05 | 17.98 | 17.98 | 17.98 | 21.57 | 21.57 |
| ^4^Average PM2,5 detection  in 2021 (ug/m3) | 8.75 | 9.27 | 9.27 | 9.27 | 13.79 | 13.79 |

NA refers to ‘non-applicable’ information.

*The closest municipality to Lleida outskirts sampling point (1.5 km apart) is Albatàrrec, which has a total population of 2,224 habitants (last update in 2021) and is located 4 km apart from Lleida city. Values indicated with * apply to this municipality.

^1^*Institut d’Estadística de Catalunya (idescat), Generalitat de Catalunya*.

^2^*Cartografia dels hàbitats a Catalunya, versió 2 (2018), Generalitat de Catalunya.* Land use/cover within a 4km-buffer around each sampling point.

^3^*Agencia Estatal de Meteorología (AEMET)* and *Institut d’Estadística de Catalunya (idescat), Generalitat de Catalunya.* Annual values and averages recovered from official annual weather maps (data obtained from *Servei Meteorològic de Catalunya, SMC*).

^4^ *Xarxa* *de Vigilància i Previsió de la Contaminació Atmosfèrica (dades obertes Catalunya), Generalitat de Catalunya*. The European Union determines that the assessment of the air quality must be done by ‘air quality zones’ because measuring every point of the territory is impossible. This system implies dividing the territory (Catalonia) into zones where pollutant levels are believed to be similar. In Catalonia, the territory is divided into 14 air quality zones (that have similar emissions and dispersion conditions), and each municipality is included in one of the 14 zones. Particular differences between the sampled localities cannot be depicted; hence, values of the closest air quality station to each locality have been illustrated in the table.

**Table S2**. **Biological data of sampled bats, time and location of sampling.**

| ID  sample | Gender | Age | Body  weight (g) | Forearm  length (mm) | Body Mass  Index | Sampling  date | Scenario | Location | X, Y coordinates |
| --- | --- | --- | --- | --- | --- | --- | --- | --- | --- |
| 01* | F | A | 6.5 | 33.30 | 0.20 | 07-2021 | D0 | St. Llorenç de Morunys | 42.136111, 1.580833 |
| 02 | M | A | 4.7 | 33.64 | 0.14 | 07-2021 | D0 | St. Llorenç de Morunys | 42.136111, 1.580833 |
| 04 | M | A | 4.5 | 32.46 | 0.14 | 07-2021 | D0 | Lles | 42.388056, 1.687222 |
| 05 | M | A | 6.0 | 35.28 | 0.17 | 07-2021 | D0 | Lles | 42.388056, 1.687222 |
| 06 | M | A | 5.5 | 33.11 | 0.17 | 07-2021 | D0 | Lles | 42.388056, 1.687222 |
| 07 | M | A | 7.0 | 32.46 | 0.22 | 07-2021 | D0 | Lles | 42.388056, 1.687222 |
| 08* | F | A | 7.5 | 34.89 | 0.21 | 07-2021 | D0 | Lles | 42.388056, 1.687222 |
| 09* | F | A | 7.0 | 34.61 | 0.20 | 07-2021 | D0 | Lles | 42.388056, 1.687222 |
| 10 | M | J | 5.5 | 32.79 | 0.17 | 08-2021 | D1 | Lladurs | 42.09256, 1.41260 |
| 11 | F | J | 5.5 | 34.25 | 0.16 | 08-2021 | D1 | Lladurs | 42.09256, 1.41260 |
| 12 | F | A | 6.0 | 35.60 | 0.17 | 08-2021 | D1 | Lladurs | 42.09256, 1.41260 |
| 13 | F | A | 6.0 | 35.25 | 0.17 | 08-2021 | D1 | Lladurs | 42.09256, 1.41260 |
| 14 | F | A | 6.5 | 33.54 | 0.19 | 08-2021 | D1 | Lladurs | 42.09256, 1.41260 |
| 15 | F | A | 6.0 | 35.00 | 0.17 | 08-2021 | D1 | Lladurs | 42.09256, 1.41260 |
| 207 | F | A | 6.0 | 35.36 | 0.17 | 09-2021 | D2 | L'Espunyola | 42.06748, 1.41260 |
| 208 | F | A | 6.0 | 33.69 | 0.18 | 09-2021 | D2 | L'Espunyola | 42.06748, 1.41260 |
| 215 | M | A | 5.0 | 33.65 | 0.15 | 09-2021 | D2 | L'Espunyola | 42.06748, 1.41260 |
| 216 | F | A | 5.0 | 32.53 | 0.15 | 09-2021 | D2 | L'Espunyola | 42.06748, 1.41260 |
| 220 | F | A | 6.5 | 34.81 | 0.19 | 09-2021 | D2 | L'Espunyola | 42.06748, 1.41260 |
| 222 | F | A | 5.0 | 32.22 | 0.16 | 09-2021 | D2 | L'Espunyola | 42.06748, 1.41260 |
| 223 | F | A | 6.5 | 34.91 | 0.19 | 09-2021 | D2 | L'Espunyola | 42.06748, 1.41260 |
| 224 | M | A | 6.5 | 36.98 | 0.18 | 09-2021 | D2 | L'Espunyola | 42.06748, 1.41260 |
| 16 | F | A | 4.5 | 34.37 | 0.13 | 09-2021 | D3 | Lleida outskirts | 41.57864, 0.64231 |
| 17 | F | A | 6.5 | 35.72 | 0.18 | 09-2021 | D3 | Lleida outskirts | 41.57864, 0.64231 |
| 18 | F | A | 6.5 | 33.88 | 0.19 | 09-2021 | D3 | Lleida outskirts | 41.57864, 0.64231 |
| 19 | F | A | 5.0 | 33.30 | 0.15 | 09-2021 | D3 | Lleida outskirts | 41.57864, 0.64231 |
| 209 | F | A | 5.5 | 35.50 | 0.15 | 09-2021 | D3 | Lleida outskirts | 41.57864, 0.64231 |
| 21 | M | A | 6.5 | 34.18 | 0.19 | 09-2021 | D3 | Lleida outskirts | 41.57864, 0.64231 |
| 217 | F | A | 5.5 | 31.61 | 0.17 | 09-2021 | D3 | Lleida outskirts | 41.57864, 0.64231 |
| 218 | M | A | 5.0 | 33.17 | 0.15 | 09-2021 | D3 | Lleida city | 41.63102, 0.64231 |
| 22 | M | A | 6.5 | 33.79 | 0.19 | 09-2021 | D3 | Lleida city | 41.63102, 0.64231 |

*Lactating females.

M: male; F: female; A: adult; J: juvenile (yearling). The age of the bats was assessed by transilluminating bat wings, showing a translucent epiphyseal cartilage of the metacarpal-phalangeal joint at the 4^th^ finger in juveniles, and rounded bony finger joint in adults. The Body Mass Index was calculated by dividing the body weight between the forearm length.

**Table S3. Pair-wise comparison of alpha and beta diversities between scenarios. Significant results are signaled with *.**

|  | **Shannon's diversity** | | | **Bray-Curtis distance** | | |
| --- | --- | --- | --- | --- | --- | --- |
|  | Kruskal-Wallis's statistic | p-value | ANOSIM's R | | p-value |  |
| D0_D1 | 33 | 0.28 | 0.15 | | 0.1 |  |
| D0_D2 | 47 | 0.13 | 0.18 | | 0.08 |  |
| D0_D3 | 64 | 0.005* | 0.28 | | 0.002* |  |
| D1_D2 | 28 | 0.66 | -0.04 | | 0.57 |  |
| D1_D3 | 37 | 0.27 | 0.22 | | 0.045* |  |
| D2_D3 | 52 | 0.14 | 0.15 | | 0.09 |  |

**Table S4**. **Biosample accession numbers for datasets obtained in this study.**

| **Biosample** | **Sample Barcode** | **Sample correspondence** |
| --- | --- | --- |
| SAMN27615841 | barcode01 | 01 |
| SAMN27615842 | barcode02 | 02 |
| SAMN27615843 | barcode04 | 04 |
| SAMN27615844 | barcode05 | 05 |
| SAMN27615845 | barcode06 | 06 |
| SAMN27615846 | barcode07 | 07 |
| SAMN27615847 | barcode08 | 08 |
| SAMN27615848 | barcode09 | 09 |
| SAMN27615849 | barcode10 | 10 |
| SAMN27615850 | barcode11 | 11 |
| SAMN27615851 | barcode12 | 12 |
| SAMN27615852 | barcode13 | 13 |
| SAMN27615853 | barcode14 | 14 |
| SAMN27615854 | barcode15 | 15 |
| SAMN27615855 | barcode16 | 16 |
| SAMN27615856 | barcode209 | 209 |
| SAMN27615857 | barcode17 | 17 |
| SAMN27615858 | barcode18 | 18 |
| SAMN27615859 | barcode19 | 19 |
| SAMN27615860 | barcode21 | 21 |
| SAMN27615861 | barcode22 | 22 |
| SAMN27615862 | barcode217 | 217 |
| SAMN27615863 | barcode218 | 218 |
| SAMN27615864 | barcode220 | 220 |
| SAMN27615865 | barcode207 | 207 |
| SAMN27615866 | barcode222 | 222 |
| SAMN27615867 | barcode223 | 223 |
| SAMN27615868 | barcode224 | 224 |
| SAMN27615870 | barcode215 | 215 |
| SAMN27615871 | barcode208 | 208 |
| SAMN27615872 | barcode216 | 216 |
